# Exploring Abnormal Behavior Patterns of Online Users With Emotional Eating Behavior: Topic Modeling Study

**DOI:** 10.2196/15700

**Published:** 2020-03-31

**Authors:** Youjin Hwang, Hyung Jun Kim, Hyung Jin Choi, Joonhwan Lee

**Affiliations:** 1 Human Computer Interaction & Design Lab Seoul National University Seoul Republic of Korea; 2 Functional Anatomy of Metabolism Regulation Lab Seoul National University College of Medicine Seoul Republic of Korea

**Keywords:** emotional eating, eating disorder, machine learning, data-driven research, behavior analysis, topic modeling, Latent Dirichlet Allocation

## Abstract

**Background:**

Emotional eating (EE) is one of the most significant symptoms of various eating disorders. It has been difficult to collect a large amount of behavioral data on EE; therefore, only partial studies of this symptom have been conducted. To provide adequate support for online social media users with symptoms of EE, we must understand their behavior patterns to design a sophisticated personalized support system (PSS).

**Objective:**

This study aimed to analyze the behavior patterns of emotional eaters as the first step to designing a personalized intervention system.

**Methods:**

The machine learning (ML) framework and Latent Dirichlet Allocation (LDA) topic modeling tool were used to collect and analyze behavioral data on EE. Data from a subcommunity of Reddit, /r/loseit, were analyzed. This dataset included all posts and feedback from July 2014 to May 2018, comprising 185,950 posts and 3,528,107 comments. In addition, deleted and improperly collected data were eliminated. Stochastic gradient descent–based ML classifier with an accuracy of 90.64% was developed to collect refined behavioral data of online users with EE behaviors. The expert group that labeled the dataset to train the ML classifiers included a medical doctor specializing in EE diagnosis and a nutritionist with profound knowledge of EE behavior. The experts labeled 5126 posts as EE (coded as 1) or others (coded as 0). Finally, the topic modeling process was conducted with LDA.

**Results:**

The following 4 macroperspective topics of online EE behaviors were identified through linguistic evidence regarding each topic: addressing feelings, sharing physical changes, sharing and asking for dietary information, and sharing dietary strategies. The 5 main topics of feedback were dietary information, compliments, consolation, automatic bot feedback, and health information. The feedback topic distribution significantly differed depending on the type of EE behavior (overall *P*<.001).

**Conclusions:**

This study introduces a data-driven approach for analyzing behavior patterns of social website users with EE behaviors. We discovered the possibility of the LDA topic model as an exploratory user study method for abnormal behaviors in medical research. We also investigated the possibilities of ML- and topic modeling–based classifiers to automatically categorize text-based behavioral data, which could be applied to personalized medicine in future research.

## Introduction

### Background

A large population is affected by eating disorders, including anorexia nervosa, bulimia nervosa, and binge eating disorder. According to statistics from the UK Addiction Treatment Centers, about 725,000 people in Britain have been affected by eating disorders, whereas others put the number as high as 1.6 million. The actual population that is affected by eating disorders is predicted to be even higher, considering the fact that many hesitate to seek professional help [1].

One of the common symptoms of eating disorders is emotional eating (EE), which is defined as “eating in response to any emotion, whether that be positive or negative” [2,3]. People with EE behavior frequently consume large quantities of comfort food that are usually nutritionally imbalanced, as a response to feelings instead of hunger [3,4]. The investigation of EE behavior is critical as many people with EE behaviors easily transition into those with serious eating disorders [5].

Few studies with a large amount of data were conducted regarding EE behavior. Analyzing the large quantity of behavioral data in diet-related posts on social media could be the first step in designing a support system for social media users. Previous studies in the field of human-computer interaction (HCI) have analyzed the users’ behaviors through the utilization of data-driven technologies [6-10]. These studies analyze various user-created contents such as texts, images, or other categories of user logs [11-13]. Meaningful information can be extracted from these data, increasing the number of studies that utilize such a large amount of data to analyze the characteristics and patterns of abnormal behaviors [14-17].

When it comes to health-related abnormalities, face-to-face personal interviews have been used as the traditional method of behavioral analysis. However, people in the predisease state rarely visit experts because of the low perceived severity of their status, and thus, it is difficult to collect the data from people in the predisease state [17]. This limited dataset can result in biased results because of the unique characteristics of the condition of each group. Moreover, data from face-to-face interaction during a short period of time are not natural data collected from daily activities [18]. Therefore, observing the subjects in their natural environment through social media scanning is a promising alternative method for abnormal behavior analysis [19,20]. Previous studies have already used the information on social media to detect depression and insomnia [21-26].

Previous studies used natural language processing (NLP) technology to recognize certain behavioral patterns in social media and determined abnormal behaviors with repeated use of keywords or synonyms in the online posts [27-30]. For example, De Choudhury et al [23] used the Reddit community data to predict individuals who are more likely to undergo transitions from mental health discourse to suicidal ideation. They analyzed the linguistic structures such as the fraction of nouns, verbs, and adverbs used in posts and comments.

In this study, we used Latent Dirichlet Allocation (LDA), which is a high-performance topic modeling tool [31-33]. Compared with traditional text analysis methods such as interpretivist text analysis and systematic qualitative coding, LDA can capture unusual structures that exist in the natural language data as it is based on unsupervised learning, which can perform more complex tasks [34].

### Objective

This study aimed to investigate the online behavior patterns of emotional eaters using the topic modeling method. We collected posts (n=185,950) and comments (n=3,528,107) from /r/loseit, a subcommunity of Reddit, an online social forum, and classified these posts and comments with a machine learning (ML) framework. LDA [32] was used to examine the behavior patterns of emotional eaters. The feedback on the EE posts was also classified into multiple topics by LDA. We also compared the different proportions of feedback topics on each behavior topic to understand the interactions in the comments section. On the basis of these findings, we discussed the design implications for a personalized supporting system in health care.

## Methods

### Data Collection

We used open-source data distributed through Google BigQuery, which originated from Reddit, one of the biggest online social news websites and forums. Contents in Reddit are organized in subcommunities by areas of interests, called *subreddits*. Among these subreddits, /r/loseit is one of the biggest subreddit community that deals with weight management. In /r/loseit, the user-generated content comprises various topics related to obesity and weight loss, such as personal experiences; recommendations; and reviews of medications, medical procedures, diets, and exercises [35]. For research ethics, to protect personal information, we did not collect any information that can be used to identify the specific users, such as personal ID and name. When including quotes in this paper, we paraphrased all sentences to remove words that can identify the individual or allow searching of the post. With this process, we constructed a dataset that included all posts and feedback between July 2014 and May 2018, comprising 185,950 posts and 3,528,107 comments. Deleted and improperly collected data were eliminated.

### Process of Data Analysis

#### Data Labeling With Expert Group

To classify data from the area of interest from the whole dataset, we trained the ML classifiers. The expert group, including a medical doctor who specializes in EE diagnosis and a nutritionist who has profound knowledge in EE behavior, labeled a large portion of the data (5126 posts) as EE (coded as 1) or others (coded as 0) to train ML classifiers. The Dutch Eating Behavior Questionnaire, the EE scale (EES) [36], the extended version of EES [37], the revised Three-Factor Eating Questionnaire [37], and the Emotional Appetite Questionnaire [38] were mainly used as reference during the labeling process. The 2 experts independently labeled 120 randomly selected posts, then discussed the labels until they reached consensus. This process was repeated 5 times. The overall labeling process yielded a Cohen kappa coefficient of 0.85. This process of human labeling was conducted based on previous works [39,40]. As a result, 563 posts were labeled as EE posts and 4563 as others (Table 1).

**Table 1:** Total number of posts used in training machine learning classifiers.

Data		Emotional eating (coded as 1)	Others (coded as 0)
**Posts used for ML^a^ classifiers, n**			
	Training set (9-fold validation)	507	4107
	Test set	56	456
	Total posts for ML	563	4563
Final posts classified by ML, n		26,154	159,796

^a^ML: machine learning.

#### Training Machine Learning Classifiers

We trained 5 kinds of ML classifiers and compared their performance in detecting EE posts. Naive Bayes, decision tree, support vector machine, k-nearest neighbor algorithm, and stochastic gradient descent (SGD) were selected to classify EE posts. To account for the imbalance caused by a low proportion of EE posts in the dataset, we considered the accuracy score as well as precision, recall, and F1 scores to evaluate the performance of the models. Among the ML classifiers, the SGD classifier showed the best performance (see Table 2). The SGD classifier achieved the highest mean accuracy (0.90), precision (0.92), recall (0.91), and F1 score (0.91). K-fold cross-validation (k=0). A mean cross-validated area under curve (AUC) of 0.89 was obtained. Receiver operating characteristic curve represented the change of true positive rate and false positive rate, whereas the AUC represents the ML model. The SGD classifier labeled 26,154 EE posts and 194,435 corresponding comments.

**Table 2:** Performance of machine learning classifiers.

Machine learning method	Precision	Recall	F1 score	Accuracy (%)
Naive Bayes	0.87	0.81	0.83	81
Decision tree	088	0.88	0.88	88
Support vector machine	0.90	0.91	0.90	91
K-nearest neighbor	0.86	0.88	0.87	83
Stochastic gradient descent	0.92	0.91	0.91	91

### Preprocessing of Data and Topic Modeling With Latent Dirichlet Allocation

To explore the online behavior patterns of emotional eaters, topic modeling process was conducted with LDA. For more advanced topic modeling, we preprocessed the EE posts classified with SGD before the LDA analysis. Natural language toolkit (NLTK) in Python was used for NLP. Preprocessing of data included the following 4 steps: (1) removing punctuation marks, (2) tokenizing and lemmatizing, (3) removing predefined stop words, and (4) performing term frequency-inverse document frequency (TF-IDF) vectorization. In step 3, removing stop words removes redundant and nonconsequential terms in the corpora. NLTK provides a built-in list of stop words, but we updated it for our research purposes. To better focus on the semantic aspects of the topics, we added auxiliary verbs and conjunctions that appeared repeatedly without particular meaning to the list of stop words (ie, “when,” “be,” “have,” “not,” “do,” “so,” “when,” “would,” “that,” “can,” and “more”). The modified list of stop words is on our Github page, with the codes of data analysis including ML classifier and LDA. This process was conducted under the supervision of experts who analyzed a subsample of terms that were considered for removal. Finally, in step 4, the texts were converted into a term-document matrix where each word was assigned a weight using the TF-IDF weighing scheme. With these preprocessed data, we conducted the LDA topic modeling.

### Statistical Analysis of Feedback Patterns

A total of number of 194,435 feedback comments on 26,154 posts were analyzed using statistical analysis. A chi-square test was conducted to distinguish the different proportions of 5 feedback topics in 4 types of EE posts.

## Results

### Topics Related to Emotional Eating

Throughout the LDA topic modeling analysis, EE posts were categorized into 4 topics: (1) EE 1, addressing feelings; (2) EE 2, sharing physical changes; (3) EE 3, sharing or asking for dietary information; and (4) EE 4, sharing dietary strategies (see Table 3). To minimize bias while categorizing the topics, the 4 topics were prudently chosen with iterative discussions among the experts, including a clinical doctor, a dietitian, and a nutritionist. All excerpts from the data were paraphrased and anonymized before being discussed.

**Table 3:** Topics of emotional eating behavior and distinguishable linguistic differences.

Number	Topic	Frequent words	Additional words found in the topic
EE^a^ 1	Addressing feelings	Feel, good, love, really, depress, food, hate, ever, much, change, and big	Defeated, disgusting, hopeless, bad, full, shit, guilty, hungry, famished, better, nice, and satisfying
EE 2	Sharing physical changes	Weight, lose, lb, pound, gain, lose, weight, get, diet, time, loss, exercise, track, lose, and scale	Shape, scalable value, nonscalable value, progress, accomplishment, regular, successful, courage, and back
EE 3	Sharing or asking for dietary information	Food, drink, meal, snack, pizza, breakfast, sugar, work, chocolate, fruit, chicken, soda, cream, chip, salad, ice, bread, and veggie	Carbohydrate, fat, protein, ideas, triggers, advice, daily, nutrition, water, experience, and favorite
EE 4	Sharing dietary strategies	Binge, think, food, eat, emotion, mental, help, binge eating, and control	Keto, calorie in/calorie out, yoyoing, couch to 5K, 1200 cal, diet, protein, extreme, and appetite

^a^EE: emotional eating.

#### User Behaviors Related to Emotional Eating Topic 1: Addressing Feelings

Topic EE 1 mainly reflects the users’ expressions of feelings toward specific eating behaviors or the food itself. The following words are sample contents analyzed based on our EE topic model: “feel,” “depress,” “good,” “love,” and “hate.” For these words, the predicted probability of EE (pEE) topic 1 (pEE 1) among 4 EE topics based on the LDA model is 100%, which means that nearly all the posts on the forum contained these words. As EE includes eating in response to any emotions, whether that be positive or negative, the appearance of positive words such as “good” and “love” in the list of frequent words is consistent with known behaviors of emotional eaters. We also discovered consistent use of words such as “defeated,” “disgusting,” and “hopeless.” This indicates that EE behaviors of EE 1 require the most delicate support, as there is a high chance that the user is currently in a sensitive emotional state. Below are example sentences that represent EE 1 behavior. pEE 1 indicates how well the sentence represents the topic EE 1:

I sit here weeping and feeling defeated... (pEE 1=0.99)

I tossed nearly all of my candy stash into the trash tonight. In fact, I threw away all my candies yesterday as well, but I went to market to get more. I’m disgusting and hopeless. (pEE 1=0.98)

#### User Behaviors Related to Emotional Eating Topic 2: Sharing Physical Changes

In topic EE 2, users shared their stories about physical changes. EE often leads to fluctuations in weight, and depending on what, when, and how much the users eat, the majority of posts in EE 2 elaborate on stories of these frequent weight changes. Linguistic features frequently used for weight units (“lb,” “pound,” “scale,” and “weight”) and the words that represent weight changes (“lose,” “loss,” “gain,” “get,” and “track”) are on the list of frequent words. The following excerpt shows the unstable physical status of the forum user with EE behaviors. Many quotes from posts contain words in the list of *additional words* of EE 2 (Table 3) such as “kilogram,” “crept up,” and “level”:

The first six months of my diet were not bad. I managed to stay under 100 kilograms, but I was determined to lose more weight. I’m awful and handling my appetite and my weight though. My weight kept creeping up and now, the last time I remember being under 100 is in February of this year. (pEE 2=0.96)

#### User Behaviors Related to Emotional Eating Topic 3: Sharing or Asking for Dietary Information

In topic EE 3, users shared or and asked for dietary information. In this topic, users mainly discussed the amount of food intake, contents of major nutrients, and calorie information. Thus, the majority of words on the list of frequent words are names of food (eg, “snack,” “pizza,” “chocolate,” “fruit,” “chicken,” “salad,” and “bread”). EE 3 behaviors are expressed as stories or lists, accompanied by the emotional status of the user. In the sample post below, the user describes his excessive eating habit as a stressful situation:

I managed to clear half of a medium-sized pizza even though it wasn’t even that good. After the first two slices, I was content, but within 5 minutes, I had to urge to eat more. I feel like I can’t bear to leave food uneaten. I kept eating until I hit four slices and finally, I restrained myself and put the other half in the freezer. (pEE 3=0.98)

#### User Behaviors Related to Emotional Eating Topic 4: Sharing Dietary Strategies

In topic EE 4, users mainly shared their dietary strategies, including diet plans and feedback on specific dietary methodologies. Most users who posted about EE 4 continued to communicate their experiences and strategies with community members. We discovered many controversial and unverified strategies shared among the users (eg, extremely restrained eating and 1200 cal diet). Frequent words in this topic seemed irrelevant to dietary strategies, but after thorough data exploration, we observed that the majority of users who posted about EE 4 questioned their own strategies and sought help or information from others. The following excerpt is a sample post for EE 4:

Last year, I pushed myself really hard and placed myself on a 1200-calorie diet while working out 5 times a week. I was always hungry and in constant pain. I lost about 30 pounds. In May, I started the keto diet with light exercising such as biking and my weight is just falling off! I find the keto diet to be very easy for me. (pEE 4=0.95)

### Feedback Analysis

In contrast to the post topics sorted based on the semantic elements of the content, feedback on the posts mainly relied on the syntax of the context. These elements reflect the linguistic characteristics of the feedback, which are meaningful information for determining the topics of the feedback. Similarly, we went through the topic modeling process without stemming out the pronouns, proper nouns, and interjections in the preprocessing stage. In the end, the topics of the feedback were categorized into 5 main topics, including (1) dietary information, (2) compliment, (3) consolation, (4) Reddit bot, and (5) health information. Frequent words and relevant examples are included in Table 4.

**Table 4:** Topics of feedback.

Number	Topic	Frequent words	Quotes from online posts
EF^a^ 1	Dietary information	Eat, food, meal, drink, breakfast, lunch, dinner, water, protein, cook, sugar, and hungry	“For a quick snack, I love to grab a stick of beef jerky from the convenience store or dollar store. Lasts a while, is fairly cheap, has between 100-150 calories, and fills me for a while”
EF 2	Compliment	Amazing, good, people, work, gift, great, job, fit, love, really, wow, congratulation, and nice	“A nice change! Inspiration for people like me going through this! :D”
EF 3	Consolation	Feel, hope, hard, lose, year, motivate, lb, month, keep, think, life, and change	“I am right there with you...But whatever, tomorrow is a new day, approach it just like you have been before this weekend. You know what you have to do and you know you can do it because you have done it before. Just put your head down and keep going!”
EF 4	Reddit bot	Post, question, answer, /r/loseit, automated, contact moderator, guide, bot, and thank	“I am a bot, and this action was performed automatically. Please [contact the moderators of this subReddit] (/message/compose/r/loseit) if you have any questions or concerns.”
EF 5	Health information	Weight, calorie, scale, loss, diet, exercise, muscle, body, deficit, count, burn, lift, and measure	“Alcohol can dehydrate you in the very short term which will show up as weight loss on the scale, but typically a body reacts by then retaining even more water until it feels that it is sufficiently hydrated.”

^a^EF: feedback on emotional eating behavior

### Feedback Distribution Based on Emotional Eating Topics

The proportions of feedback topic distribution differed greatly depending on the topic of the post (Multimedia Appendix 1). Table 5 lists the dominant EF topic for each EE topic. A chi-square test of feedback topics among the 4 EE behaviors revealed statistically significant results. For the posts addressing feelings (EE 1), feedback sharing dietary intake (EF 1) were dominant. EE posts regarding physical changes (EE 2) were accompanied by compliments (EF 2) on daily achievements. EE posts sharing or asking for dietary information (EE 3) collected the most significant proportion of feedback with consolation. EE posts sharing dietary strategies (EE 4) were followed by feedback with health-related information.

**Table 5:** Dominant feedback topic on each emotional eating–related behavior topic.

EE^a^ topic	Dominant EF^b^ topic	Ratio of feedback dominance	Chi-square value (*df*)	*P* value
EE 1: addressing feeling	EF 1: dietary information	0.49	28328 (4)	<.001
EE 2: sharing physical changes	EF 2: compliment	0.46	19133 (4)	<.001
EE 3: sharing or asking for dietary intake	EF 3: consolation	0.34	1813.2 (4)	<.001
EE 4: sharing dietary strategies	EF 5: health information	0.30	3181.1 (4)	<.001

^a^EE: emotional eating.

^b^EF: feedback on emotional eating behavior.

## Discussion

### Data-Driven Approach for the Determination of Users’ Emotional Eating Behavior

This study introduces a data-driven approach for determining the abnormal behaviors (ie, EE) of social forum users. EE-related data were classified with our trained SGD classifier, and 4 types of EE behaviors and 5 types of feedback were distinguished with the LDA topic modeling method. The proportions of the feedback topics significantly differed for each EE behavior topic.

#### Posts Addressing Feelings

Previous studies have proven that both positive and negative emotions can lead to EE behaviors [3,36,41]. According to our Table 3, there were many words related to feelings such as depression, anger, and joy. Depression is an incessantly occurring feeling before and after EE [41]. Emotions such as anger, fear, sadness, and joy often last a long time and linger [42,43], which could explain the frequent usage of these words in posts that address feelings. Low-arousal states such as boredom and depression are often associated with inhibition of food intake, especially compared with high-arousal states such as tension and fear. However, depression was at the top of the list of frequent words for emotional eaters, so we inferred that depressive feelings frequently occur with EE behavior [2,41]. This accumulation of emotional data allows us to analyze what provokes EE behavior and guides us to implementing a feedback function as a personalized support system (PSS).

#### Posts Sharing Physical Changes

Users with EE behaviors share their physical changes not only as a way to monitor themselves but also with expectations of social support according to our data. Thus, it is not surprising that the most dominant feedback on this type of post is a compliment. We believe that sharing physical changes through the online community can be a useful tactic for emotional eaters to get encouragement. In addition, we can track the users’ health status and design a PSS that provides behavioral guidelines in response to the users’ physical changes.

Interestingly, some of the people who showed the behavior of sharing their physical changes had obsessive characteristics (eg, weighing too often and reacting sensitively to small changes in the body). This obsessive nature often forces them to implement an overly strict plan that restrains eating, which leaves them more vulnerable to EE in response to stressful situations [44,45]. Therefore, the PSS could include a warning that extreme dietary restrictions may interfere with long-term dietary management and may even interfere with weight control.

#### Posts Sharing or Asking for Dietary Information

It is not easy to predict the dietary patterns of emotional eaters because EE occurs unpredictably. In the field of medicine or nutrition, the food frequency questionnaire or 24-hour recall methods are used to track dietary information [46]. However, the dietary information collected through these methods often lack representation because of insufficient data or problems with memory retrieval [46]. Researchers have tried to improve these dietary tracking methods with direct inputs of food intake, but these methods are cumbersome for long-term use [47]. Many social forum users share their dietary information through social media such as Twitter and Instagram [48,49]. Social media offers users with better and easier experiences in terms of recording dietary information. Therefore, this study takes advantage of the opportunity to use the dietary information on online communities for analysis.

#### Posts Sharing Dietary Strategies

Through posts sharing dietary strategies, we were able to identify diets such as calories in-calories out, keto, and 1200 kcal diet strategies. We also discovered that these dietary strategies can pose a serious health threat or aggravate EE behavior among emotional eaters [5]. As these strategies are contentious, the feedback sharing health information (EF 5) was the majority of the feedback on posts about dietary strategies. People with EE behavior should abstain from following controversial dietary strategies, but if they were to adhere to such diet, they should be well aware of the side effects such as appetite fluctuations and malnutrition [50]. It is critical that reliable information is shared among those with abnormal health behaviors as false information posted on an online community can cause damage to a large group of people. However, studies have shown that 89% of health-related information provided on online medical forums was written by people without professional experience [51,52] or medical practices [53,54]. By providing tailored information complied by experts and reliable references, PSS can be a crucial solution to false health information and discussions in the online community.

### Feedback Topics

Previous studies have highlighted the importance of feedback on health-related behaviors [55]. Feedback analysis revealed that feedback differed greatly depending on the EE behavior patterns. Feedback on EE behavior (EF) were categorized into 5 topics by experts based on LDA results (see Table 3). Dietary information in EF 1 describes not only personal dietary experiences but also dietary facts, both with and without proper reference. Compliments in EF 2 is one of the most effective forms of feedback [55] that keeps users motivated for a long period [56]. Consolation in EF 3 differs from a compliment as consolation feedback mostly appears on the posts of negative status [57] and aims to uplift those in challenging circumstances. Bot-generated feedback (EF 4) was easily distinguishable from other feedback, so the LDA model was able to classify them with high performance. EF 5 contains health-related information.

### Latent Dirichlet Allocation Topic Model as a User Study Method Before Designing a Support System for Users With Abnormal Behaviors

One of the challenges of user study comes from repeated experiments without proper understanding of user characteristics and preparation procedures. Thus, a long-standing investigation of previous studies and a pilot study attempt to overcome this challenge. Nevertheless, exploration of user characteristics through these methods still faces limitations as pilot studies can only reflect the characteristics of a limited subgroup. This gap may be ignored in small group studies, but it will lead to a significant difference in large studies.

Although the topic modeling method does not completely overcome the limitations of prior methods, it can be useful to user groups with abnormal behaviors. In addition, through topic modeling, we were able to identify prior systems that users with EE relied on (eg, MyFitnessPal for physical changes [EE 2] and total daily energy expenditure for dietary information [EE 3]). Therefore, topic modeling is applicable as a good alternative data-driven qualitative method for designing a support system for specific user group.

From the HCI-based approach, we propose a systematic design for a PSS that provides reliable and confidential information just-in-time. Although there are many health-specific websites that offer focused information verified by professionals, many online users prefer to discuss health issues on social media [58]. Therefore, it is also necessary to conduct additional research on designing social media–based PSS as a way to support the users on social media.

Previous studies demonstrate the need of personalized interventions that are customized and predetermined [59,60]. This study contributes to the medical field by detecting and analyzing abnormal behavior patterns of emotional eaters who are in need of PSS design. In addition, the ML classifier used in this study is highly applicable in the PSS development process as it can detect EE-related posts just-in-time with high accuracy.

### Limitations

This study was the first step toward understanding abnormal behavior patterns of EE to design PSS. Thus, there were several limitations that can be improved in the future works.

First, although we used a systematic topic modeling method in a data-driven approach to explore EE behavior, the categories of the topics were determined empirically by humans. In this study, we gathered domain experts to discuss their opinions and relied on their decisions to compensate for the limitations of methods. The researchers did not intervene in the decision-making process after providing the experts with sufficient explanations on the topic modeling method. Further research on establishing a standard procedure for feature selection, especially the number of categories of post topics, is necessary. In this study, EE behaviors were only sorted into 4 different categories to observe the macroscopic patterns of behaviors. However, for a more advanced system design, EE-related posts should be further distinguished into smaller subcategories with clear classification criteria.

Next, we discussed the need for PSS based on the results of our study. However, before we establish a support system, we need to investigate how users will react to bot intervention and whether this intervention will increase the users’ engagement in the community. The roles of community bot have been debated recently among the HCI community [61-63], but further research, specifically regarding users with abnormal behaviors, needs to be conducted.

Finally, as the EE data were collected from a single Reddit subcommunity dealing with weight management, there can be a bias that classifies the posts as EE behavior. However, we have attempted to minimize this bias through sufficient discussion among experts during the process of data classification and excluded posts that were not relevant to EE.

### Conclusions

This work investigates the behavior and feedback patterns of Reddit users with EE behaviors. First, we analyzed the data classified with our ML classifier to detect EE behaviors in the online community. Second, we analyzed EE behaviors and feedback topics with LDA. EE behaviors were classified into 4 main topics: addressing feelings, sharing physical changes, sharing dietary information, and sharing strategies to control EE. EF behaviors were classified into 5 topics: dietary information, compliment, consolation, Reddit bot, and health information. Our work significantly extended prior user studies on abnormal behavior patterns in the field of digital medicine research. Furthermore, our results provide new insights for designing a PSS for users with abnormal behaviors. The main contributions of this work are as follows:

- ML classifier with high accuracy that collects behavioral data of posts demonstrating EE behaviors,
- Possibility of the LDA topic model as an exploratory behavioral research method for classifying abnormal behaviors in the field of digital medicine,
- Opportunities for PSS implementation to help emotional eaters.

## Appendix

Multimedia Appendix 1 Feedback topic proportion depending on the post topic.

## Abbreviations

AUC: area under curve
EE: emotional eating
EES: emotional eating scale
EF: feedback on EE behavior
HCI: human-computer interaction
LDA: Latent Dirichlet Allocation
NLP: natural language processing
NLTK: natural language toolkit
pEE: probability of emotional eating
PSS: personalized support system
SGD: stochastic gradient descent
TF-IDF: term frequency-inverse document frequency
