# Supplementary figures and images for: Exploring Abnormal Behavior Patterns of Online Users With Emotional Eating Behavior: Topic Modeling Study
Source: J Med Internet Res. 2020 Mar 31;22(3):e15700. doi: 10.2196/15700 (PMC7157499; doi:10.2196/15700)

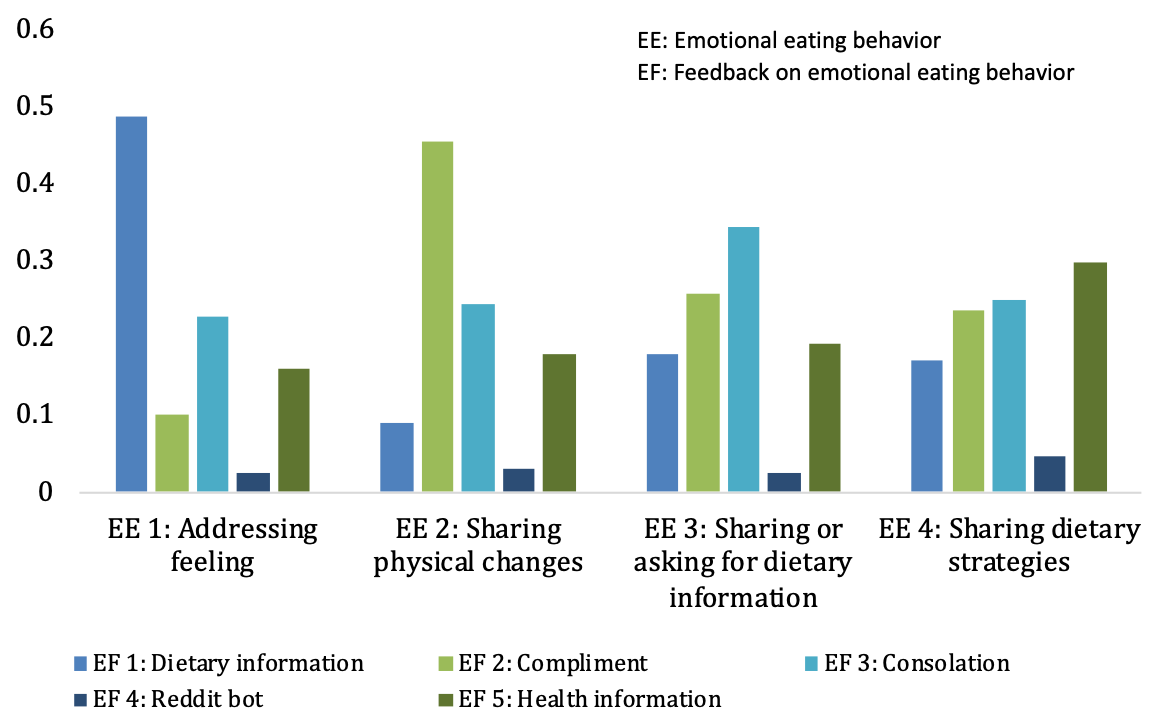

Supplement: Multimedia Appendix 1 [file jmir_v22i3e15700_app1.png]
